# Effect of pneumatic and cold compression on muscle performance and recovery in combat sports athletes

**DOI:** 10.1038/s41598-025-29014-1

**Published:** 2025-11-22

**Authors:** Robert Trybulski, Sebastian Klich, Juan Antonio Valera-Calero, Adam Kawczyński, Cesar Fernández-de-Las-Peñas, Adrian Kużdzał

**Affiliations:** 1 Medical Department Wojciech Korfanty, Upper Silesian Academy, Katowice, Poland; 2 Provita Żory Medical Center, Żory, Poland; 3 Department of Sport Didactics, Wrocław University of Health and Sport Sciences, Wrocław, Poland; 4 Department of Radiology, Rehabilitation and Physiotherapy, Complutense University of Madrid, Madrid, Spain; 5 Grupo InPhysio, Instituto de Investigación Sanitaria del Hospital Clínico San Carlos (IdISSC), Madrid, Spain; 6 Faculty of Medicine, Wrocław University of Science and Technology, Wrocław, Poland; 7 Department of Physical Therapy, Occupational Therapy, Physical Medicine and Rehabilitation, Universidad Rey Juan Carlos, Madrid, Spain; 8 Institute of Health Sciences, University of Rzeszów, Rzeszów, Poland

**Keywords:** Musculoskeletal system, Muscle

## Abstract

This study investigated the comparative effectiveness of three recovery interventions—pneumatic intermittent compression therapy (PICT) at pressures of 25 mmHg and 100 mmHg, cryo-compression therapy (CCT), and passive rest—on muscle recovery following exercise-induced fatigue in professional combat sports athletes. The primary aim was to determine which method better facilitates physiological and perceptual recovery after a fatigue protocol typical for combat sports demands. Forty-eight highly trained male and female athletes aged 18 to 40 years, each with a minimum of three years’ combat training experience, participated in this randomized controlled trial. Participants were allocated equally into four groups corresponding to the recovery modalities. The fatigue protocol involved repeated maximal plyometric box jumps until exhaustion to induce muscular stress and damage. Recovery interventions were administered immediately post-exercise, and at 24 and 48 h thereafter. Measurements were recorded at baseline, immediately post-fatigue, 30 min, and 48 h following the recovery intervention. Physiological outcomes included tissue perfusion assessed via laser Doppler flowmetry, muscle elasticity measured with a myotonometer, serum lactate dehydrogenase (LDH) activity as a marker of muscle damage, reactive strength index (RSI) obtained from jump tests, and pressure pain threshold (PPT) to evaluate soreness. Data analysis revealed significant improvements in tissue perfusion and muscle elasticity with both pneumatic compression and cryo-compression therapies when compared to passive rest. Notably, PICT at 100 mmHg maintained superior muscle elasticity up to 48 h post-exercise, while CCT produced a more immediate reduction in muscle soreness. LDH activity increased across all groups following fatigue, reflecting muscle damage. RSI decreased initially but showed differential recovery patterns depending on the intervention. In conclusion, compression therapies demonstrate clear benefits in accelerating recovery processes through enhanced blood flow and improved muscle mechanical properties, with pressure magnitude influencing outcomes. The findings support the clinical application of targeted compression strategies to optimize recovery in combat athletes, potentially improving performance and reducing injury risk. Further studies should explore long-term effects and integrate additional functional and biochemical parameters to refine rehabilitation protocols.

## Introduction

Combat sports athletes frequently experience high levels of muscular stress and are often subjected to eccentric contractions during fights, which can significantly increase muscle stiffness and pain^1^.Especially, Mixed Martial Arts (MMA) fighters who adopt a “striker” style are particularly susceptible to these muscular effects, as their training and competition involve numerous low and high kicks, defense techniques, wrestling maneuvers, throws, and jumps. The excessive load on skeletal muscles during such intense activity can lead to mechanical damage to cell membranes, activation of inflammatory processes, increased muscle soreness, stiffness, and reduced elasticity. Effective muscle tissue regeneration after intense exercise is crucial for the continued performance of the athletes^2^.

Various factors, such as muscle inflammation, nitric oxide signaling, and mechanical stress influence regenerative processes. Depending on the specific circumstances and timing, these factors can either promote or hinder regeneration^3^. Additionally, satellite cells, specialized progenitor cells located near muscle fibers, play a crucial role in muscle repair and growth. When activated in response to muscle injury or overload, these cells proliferate and differentiate into myoblasts, which ultimately fuse to repair damaged muscle fibers or contribute to muscle hypertrophy^3,4^. Therefore, a complex relationship between exercise-induced muscle damage and recovery has attracted considerable attention in sports medicine, particularly in the search for effective physiotherapy methods^5^.

Among various recovery methods and strategies, pneumatic intermittent compression therapy (PICT) has emerged as an effective intervention to improve muscle recovery after strenuous physical activity^6^. PICT is based on the application of rhythmic pressure to peripheral tissues, theoretically facilitating improved blood flow, reducing inflammation, and accelerating waste removal^7^. Additionally, PICT uses a series of compression cycles to enhance blood flow and tissue perfusion. By using inflatable sleeves that rhythmically inflate and deflate, PICT generates external pressure that stimulates the venous and lymphatic systems. This process facilitates waste removal and increases oxygen delivery to muscle tissues^6,7^. On the other hand, cryo-compression therapy (CCT) is commonly used as an alternative recovery strategy^8,9^. This method is based on a cold stimulus to tissue with a certain pressure through a calf. The pressure may vary but ranges from 15 to 75 (mmHg) (2–10 kPa), and the temperature is from 3 to 45 °C. The duration of this treatment varies from 10 to 30 min^8^. PICT is emerging as a potential benefit to natural mechanisms by facilitating increased blood flow and nutrient delivery to muscle tissues^10^, which may further optimize the recovery environment and reduce excessive muscle stiffness and soreness following intense exercise^4^. Such intervention may reduce inflammation and enhance cell signaling pathways critical for muscle repair, reducing the risk of injury and influencing athletic performance^11^.

Understanding physiological mechanisms is essential to developing effective recovery strategies and may improve athletic performance. Currently, studies focused on muscle recovery using PICT have not established a clear research perspective. Many studies have small sample sizes, which reduce the statistical power necessary to draw definitive conclusions about the effectiveness of these interventions^6,12,13^. Additionally, inconsistent treatment protocols, such as varying compression duration, frequency, and pressure settings, contribute to the variability of results and hinder direct comparisons between studies^4,10^. To address this issue, further research is needed to establish guidelines for PICT therapy. Furthermore, the reliance on subjective measures of muscle soreness or fatigue raises concerns about the objectivity and reliability of findings, emphasizing the need for objective measures to evaluate the efficacy of PICT. These limitations highlight the need for further research with gaps in the methodologies to evaluate the role of pneumatic compression, specifically using innovative techniques such as CCT in the improvement of athletes’ muscle recovery. Consequently, there is a need for research to evaluate recovery monitoring technologies to better assess the efficacy of recovery interventions. Recent studies have reported the potential of technological tools, such as wearable devices, to provide precise and objective assessments of recovery status after exercise^14^. Moreover, investigations into warm-up protocols and ergogenic aids in combat sports demonstrate how these strategies can significantly influence physical performance, perceived exertion, and psychological readiness^15^. Additionally, research on repeated sprint ability highlights the critical role of recovery time in maintaining performance across high-intensity bouts in sports requiring quick recovery, such as soccer, which may be extrapolated to combat sports scenarios^16^. Therefore, this study aimed to compare the effect of different recovery strategies on physiological outcomes, including muscle damage indicators after a fatigue-induced protocol. We hypothesized that the PICT recovery strategy may be particularly useful in MMA fighters, as a result of the greater impact of eccentric contractions on muscle damage and acute inflammation.

## Materials and methods

### Study design

This study used an experimental, prospective, randomized, controlled study design conducted between September 2023 and December 2023 in the Provita Medical Center. The RCT was registered in the ISRCTN registry under the number ISRCTN90040217 (first registration date: 25/05/2023). The study was approved by the ethics committee of the National Council of Physiotherapists (approval code: 9/22; approval date: 6 April 2022) and conducted by the Declaration of Helsinki. Participants were assigned to one of four intervention groups, including pneumatic compression therapy (PICT) at (1) 25 mmHg and (2) 100 mmHg, (3) cryo-compression therapy (CCT), and (4) control group. Each participant was measured at the following time-points, i.e., at baseline, immediately after the fatigue procedure, 30 min, and 48 h after recovery strategies. Before the initial assessment, all participants were randomly assigned to one of the three experimental groups or control group (passive rest). The fatigue protocol consisted of repeated plyometric jumps until exhaustion. The study assessed multiple outcome measures, including physiological markers (perfusion [nonreference perfusion unit], elasticity [NaN], lactate dehydrogenase activity (LDH) [U/L], and reactive strength index (RSI) (m∙s^−1^) and perceptual markers of recovery (pressure pain threshold (PPT) [N/cm]).

### Subject

The required sample size was estimated using G*Power 3.1.9.2 (Kiel University, Germany), based on a repeated measures ANOVA with four groups and four time points. A total sample of 44 participants was determined to achieve 90% power (α = 0.05, effect size f = 0.25, correlation among repeated measures = 0.5). To allow for potential dropouts, 48 athletes were ultimately included (12 per group).

Forty-eight professional male (n = 29) and female (n = 19) professional combat sports athletes were involved in this study. Each experimental and control group consisted of twelve participants. Detailed characteristics, including anthropometric parameters and information on the participants’ training experience, are presented in Table 1. Each of the participants met the following inclusion criteria: (1) age 18–40 years; (2) at least 3 years of experience in combat sports training; (3) training at least five times per week. Considering McKay’s participant classification scheme, the study participants were at level 3: highly trained/national level^14^. In addition, athletes declared they participated in at least one motor training session per week, which included plyometric exercises. The exclusion criteria were as follows: (1) elevated blood pressure before the study (blood pressure > 140/90 mm Hg); (2) currently treated injuries, damaged skin, or unspecified skin lesions at the measurement sites; (3) tattoo at the measurement site (as this interfered with tissue perfusion measurements); (4) taking any medication, including painkillers and hormonal agents. Tattooed skin was excluded from measurements due to potential interference with laser Doppler signal caused by pigment absorption properties. Athletes were also excluded in case of extreme fatigue, fever, infection, or at the explicit request of the participant at any time during the study. Written informed consent was obtained from the participants after they were informed of the study conditions. Participants were required to refrain from training for 24 h before and 48 h during the study. In addition, due to tissue perfusion measurements, participants were asked to refrain from consuming any ergogenic drinks (a list of excluded products was provided to participants) for 4 h before the study.

**Table 1:** Mean ± SD of the participant characteristics.

Variables	PICT at 25 mmHg	PICT at 100 mmHg	CCT	Control
Age (year)	24.6 ± 3	28.5 ± 6	26.0 ± 4	29.0 ± 5
Sex Body height (m)	♂: 10 ; ♀: 2 1.78 ± 11	♂: 10 ; ♀: 2 1.77 ± 6	♂: 9; ♀: 3 1.75 ± 9	♂: 9 ; ♀: 3 1.76 ± 10
Body mass (kg)	71.1 ± 12	80.0 ± 13	78.0 ± 15	80.0 ± 11
Training experience (years)	7 ± 2	11 ± 3	10 ± 4	11 ± 6

### Randomization

**Fig. 1:**
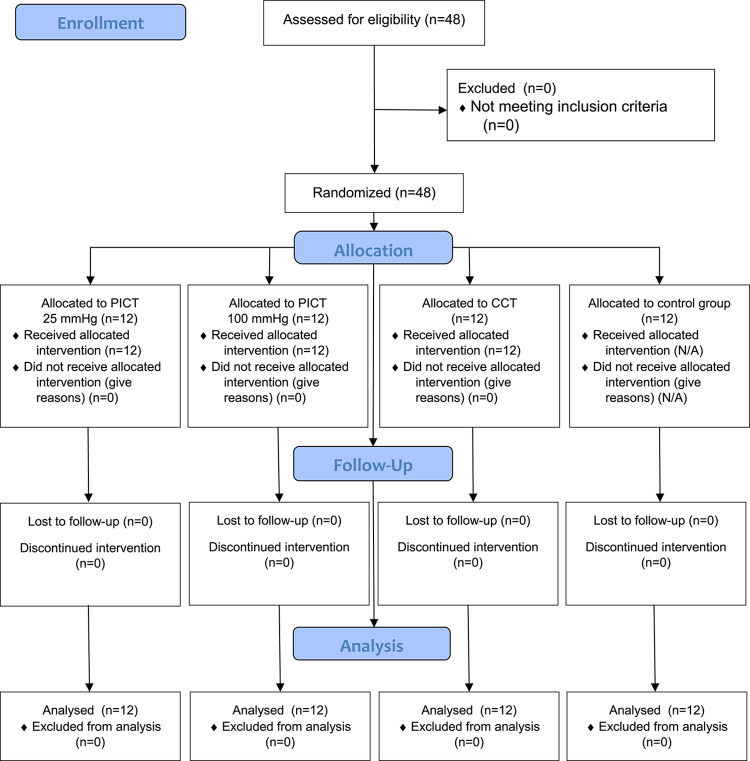
Enrolment, randomization, and dropout of participants allocated to the intervention group (pneumatic compression therapy (PICT) at (1) 25 mmHg and (2) 100 mmHg, (3) cryo-compression therapy (CCT) and (4) control group.

Group allocation was conducted using simple 1:1 randomization via an online sequence generator (www.randomizer.org). Although outcome assessors were not formally blinded, all measurements were conducted using objective, standardized protocols to minimize observer bias. In addition, randomization determined the session in which each participant received the intervention. One week before the start of the study, participants completed a familiarization session, which included 10 min of CCT stimulation and a 30-s bout of plyometric jumping to standardize exercise technique (Fig. 1).

### Interventions

Participants were randomly assigned to one of three intervention groups:

1. Pneumatic compression therapy (PICT) at 25 mmHg.

**Fig. 2:**
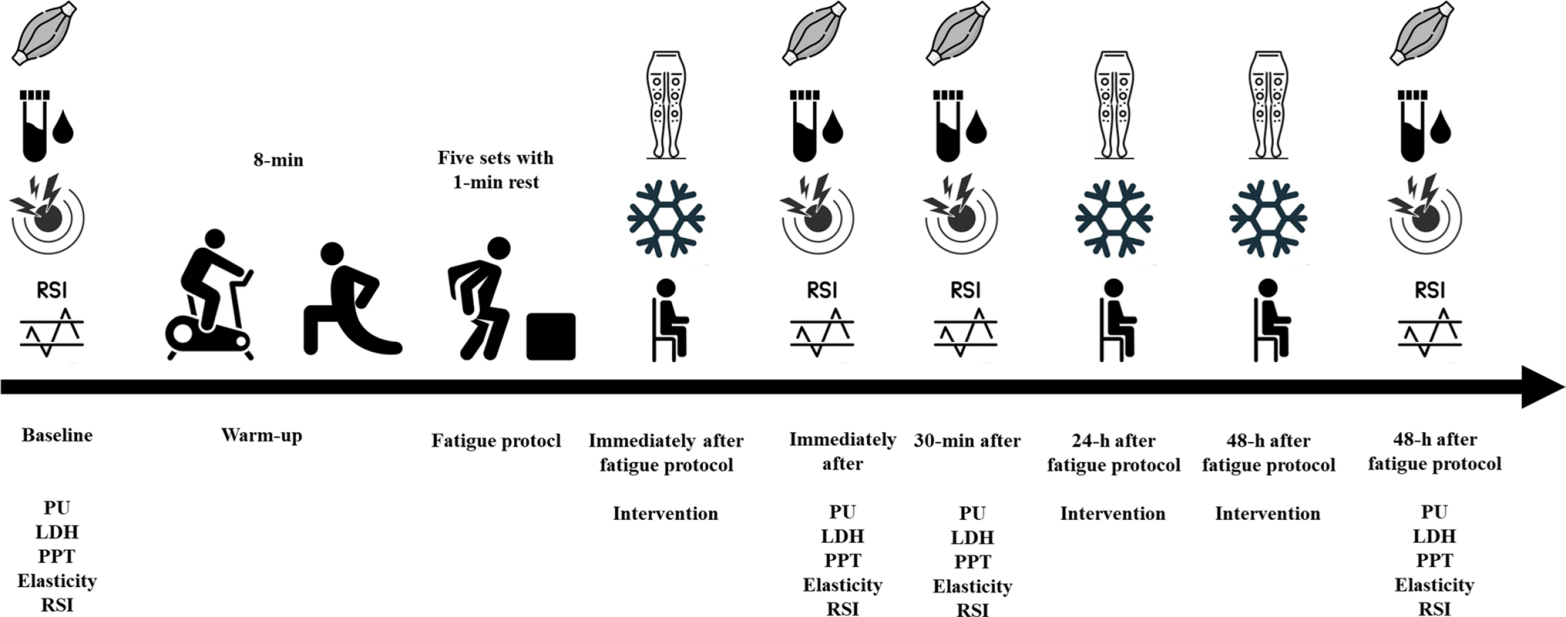
The experimental procedure included tissue perfusion (PU), lactate dehydrogenase activity (LDH), Pressure pain threshold (PPT), muscle elasticity, and Reactive Strength Index (RSI) at baseline and immediately post, 30 min, and 48 h after the fatigue protocol.

**Fig. 3:**
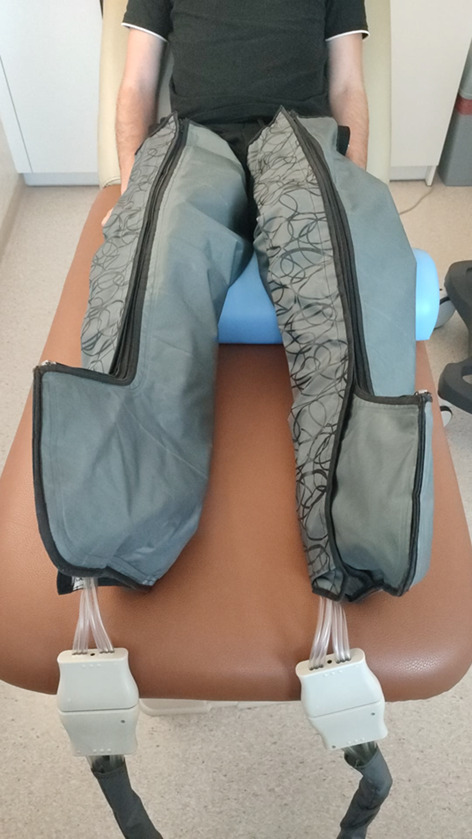
Alternating pneumatic compression therapy.

For the PICT at 25 mmHg group, participants underwent three sessions of a regenerative intervention, each lasting 20 min. (Fig. 2) During each session, a pneumatic compression device, equipped with manikins was applied to both extremities. The cuff pressure alternated between 15 and 25 mmHg every minute^15^ (Fig. 3).

2. For the PICT at 100 mmHg group, the recovery strategies were similar to 25 mmHg. However, the cuff pressure alternated between 75 and 100 mmHg every minute (Fig. 3).

3. Cryo-compression therapy (CCT) was performed with a cuff pressure of 75 mmHg (maximum capacity of the device) and a temperature of 5 °C. No pneumatic pressure variation was used in this group.

**Fig. 4:**
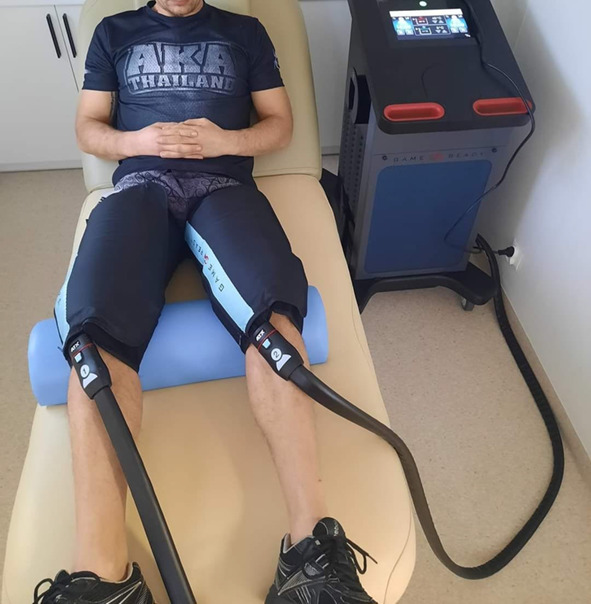
Cryo-compression therapy.

The CCT group underwent three sessions of regenerative intervention cryo-compression alternating therapy, each lasting 20 min, at a pressure of 75 mmHg (the maximum for this device) and a temperature of 5 degrees Celsius (Fig. 4).

4. Participants in the control group remained in a seated passive rest position for the same duration (20 min) as the intervention groups, immediately following the fatigue protocol.

### Experimental procedure

All participants from the experimental groups (both PICT and CCT) underwent three recovery sessions: (1) immediately after, (2) 24 h, and (3) 48 h after the fatigue protocol. Participants in the control group were instructed to refrain from any recovery-related activities (e.g., stretching, massage, cryotherapy) during the 48-h study period. Only the dominant leg (right) was used for measurement purposes. The control group remained in passive rest. Leg dominance was determined based on participants’ self-reported preferred leg used for kicking. Measurements were taken at (1) baseline, (2) immediately after fatigue protocol (after the first recovery session), (3) 30 min, and (4) 48 h after recovery procedures (after the third session) (Fig. 2). All measurements were performed in the same standardized supine position with hip and knee joints straight on a medical couch. The research and tests were conducted at the same time, 8–12 am, in the Provita Medical Center. During the research, a paramedic watched over the health of the participants. The same conditions prevailed during the tests: air temperature 21 °C and air humidity 45–50%).

### Measurements

#### Anthropometric characteristics

The Accuniq BC720 body composition analyzer (South Korea 2019) was used to assess the anthropometric characteristics of the study participants. Measurements were taken at a single point in the medial part of both rectus femoris muscles under ultrasound guidance (Sonoscape E2, China) to locate the widest cross-sectional area of the rectus femoris muscle (RF)^1^. This location was marked with a marker.

#### Tissue perfusion

Transcutaneous measurement of rectus femoris muscle perfusion was measured at rest and after stimulation using laser Doppler flowmetry (LDF) with a Perimed device (Sweden, 2004). LDF, a gold standard for assessing microcirculation, offers high sensitivity and repeatability^16^. Measurements were taken at a skin tissue volume of 1 mm^3^ and a depth of 2.5 mm. The LDF method’s repeatability, high sensitivity, and non-invasive nature enables precise assessment of microcirculation changes in response to physical stimuli^17^. Well-established measurement standard guidelines were followed^18^. Measurements were taken for 2 min. Some authors suggest that skin hyperemic reactions can be indicative of muscle tissue changes^19^. When examining tissue perfusion, “PU(r)” (resting perfusion) and “PU(p)” (post-stimulation hyperemia) are commonly assessed.

#### Muscle elasticity

A hand-held myotonometer device (MyotonPRO AS, Myoton Ltd, Estonia 2021) was used to evaluate muscle elasticity. Muscle elasticity was quantified using logarithmic decrement (unitless), as provided by the MyotonPRO device. For context, typical values for the rectus femoris range between 1.0 and 1.4 in resting athletes.The examiner positioned the probe at a right angle to the area being tested. The probe then emitted three pulses, which were applied to the testing area. Participants were positioned on their backs with their feet resting on the massage table. The elasticity of the rectus femoris muscle was measured at the muscle–tendon junctions, both proximal to the muscle belly^20^.

#### Lactate dehydrogenase activity (LDH)

Blood samples for serum enzyme activity testing (8 ml in total) were collected from the antecubital vein in the sitting position by puncture into Innmedis tubes (Poland) by qualified personnel. The tubes were immediately sent to the laboratory, where the blood was centrifuged (3000 g for 10 min at 4 °C) using a centrifuge (MPW -54). The serum was transferred to tubes and stored at − 80 °C until analysis. A certified laboratory diagnostician analyzed all samples to minimize the influence of inter-assay variability. Dehydrogenase activity was determined in serum by colorimetric spectrophotometry at 340 nm and 37 °C using ready-made diagnostic reagents (Alpha Diagnostic, Poland) in an automated biochemical analyzer A15 (Randox, Poland)^9^.

#### Reactive strength index (RSI)

The RSI was assessed using a Force Decks ground reaction force plate (Vald-Performance, Australia, 2012), a reliable and repeatable tool^21^. RSI reflects an individual’s ability to rapidly transition from eccentric to concentric muscle contractions, providing insights into reactive strength. Participants performed a maximum vertical jump from a 50 cm box onto the force plate^1^.Before each RSI measurement, except for the after-fatigue assessment, participants completed a 5-min warm-up consisting of 3 min of stationary cycling and leg stretching exercises (e.g., leg swings, high knees, arm circles). A research assistant guided the participants through the jumping technique, allowing for three practice attempts before the official measurement^22^. The best performance was used for analysis. RSI was calculated using the formula: RSI = Jump height/Contact time (m∙s − 1)^23^.

#### Pressure pain threshold (PPT)

The FPIX algometer (Wagner Instruments, Greenwich, CT, USA, 2013) was used to objectively assess pain sensitivity^24^. Measurements were taken on the vastus medialis (VM) and rectus femoris (RF) muscles, following the manufacturer’s instructions (Wagner Instruments). A pressure probe (r = 4 mm) was applied to a specific marked area on each muscle, and compressive force was gradually increased until the participant signaled discomfort^25^.The average of three measurements was recorded as the PPT value.

#### Exercise intervention

Due to the complex nature of combat sports, comprehensive performance assessment remains challenging. However, evaluating specific physiological and physical characteristics, such as those related to plyometric ability, can provide valuable insights into an athlete’s potential for success. Plyometric training, which involves explosive exercises that combine eccentric and concentric muscle actions, has been widely recognized as an effective training method for combat sports athletes^26,27^. To induce fatigue and muscle damage, we employed a plyometric jump protocol consisting of five sets of maximum effort box jumps from a 50 cm box, with a 1-min rest period between sets^1^. Each jump involved the following phases: initial phase, jump phase, load reaction phase on the box, initial phase of the landing jump, dynamic jump, and load reaction phase on the ground. Before the fatigue protocol, participants completed a 5-min warm-up of moderate-intensity cycling and 3 min of lower limb stretching. Throughout the protocol, experienced physiotherapists monitored participants to ensure proper technique and safety.

#### Statistical analyses

Data were analyzed using SPSS 25 statistical software (SPSS Inc., Chicago, Illinois, USA). Mean values and standard deviations were calculated for all variables. The Shapiro–Wilk test confirmed that the data were normally distributed and Levene’s test showed homogeneity of variances. A 4 × 4 mixed-model ANOVA (Group × Time) was used to analyze physiological and perceptual outcomes. Post-hoc pairwise comparisons were adjusted using the Bonferroni correction. Effect sizes were reported using partial eta squared (η^2^), interpreted as small (0.01–0.05), medium (0.06–0.13), or large (≥ 0.14).

## Results

### Tissue perfusion

**Fig. 5:**
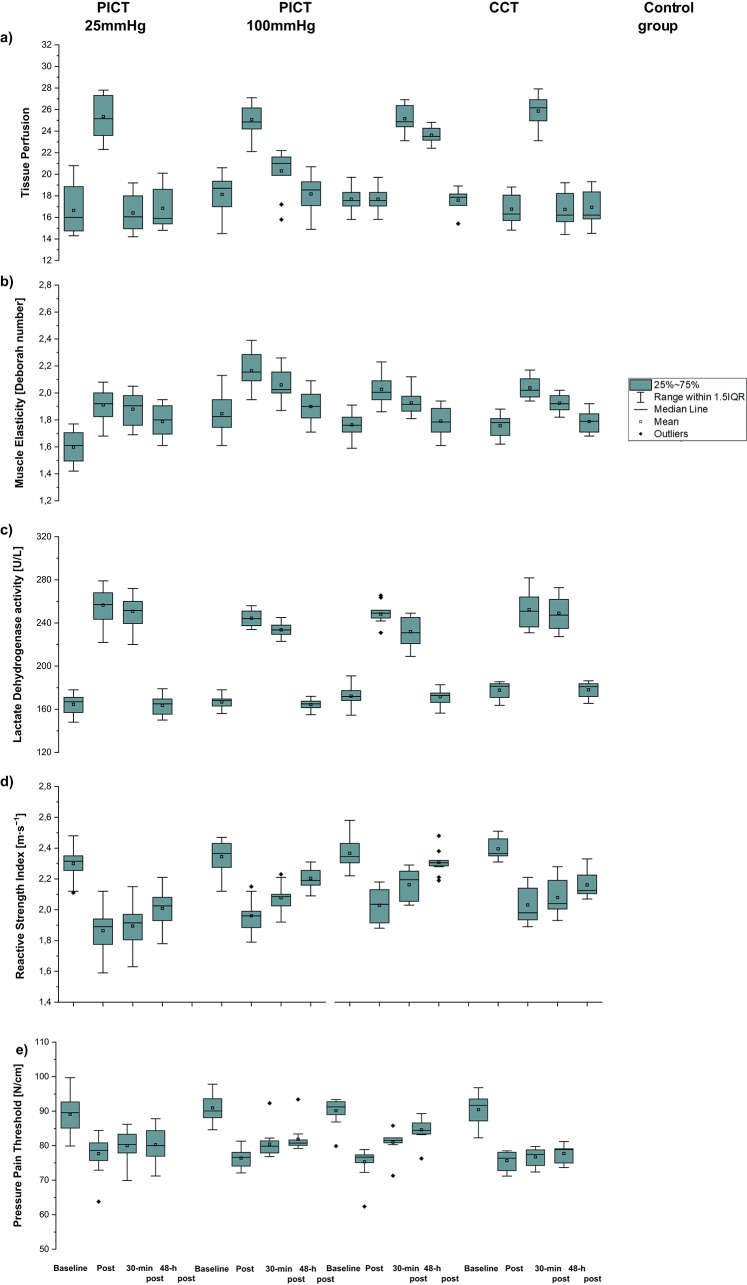
Mean ± SD of tissue perfusion (**a**), muscle elasticity [Deborah number] (**b**), lactate dehydrogenase activity (LDH) [U/L] (**c**), reactive strength index (RSI) (m∙s^−1^) (**d**), and pressure pain threshold [N/cm] (**e**) after three different recovery strategies (pneumatic compression therapy (PICT) at 25 mmHg and 100 mmHg, cryo-compression therapy (CCT) and control group) at baseline, immediately after (Post), 30-min, and 48-h after fatigue protocol.

Figure 5a shows the mean ± SD of tissue perfusion in both lower extremities at baseline, immediately after, 30 min, and 48 h post-recovery. The mixed ANOVA revealed significant main effects of *Intervention* (F_3,264_ = 1207.0, *p* < 0.001, η^2^ = 0.93) and *Time* (F_3,68_ = 14.0, *p* < 0.001, η^2^ = 0.32), as well as a significant interaction between *Intervention* × *Time* (F_3,69_ = 23.4, *p* = 0.001, η^2^ = 0.44) on tissue perfusion. Post-hoc analysis showed that tissue perfusion increased from baseline to immediately and 30 min after fatigue protocol in CCT and PICT at 100 mmHg (*p* < 0.001 for both), and from baseline to immediately after fatigue protocol in PICT at 25 mmHg and the control group (p < 0.001 for both). Additionally, tissue perfusion was greater in CCT compared to other recovery strategies at 30 min after fatigue protocol (*p* < 0.001, all). PICT at 100 mmHg showed greater tissue perfusion compared to the control group at 30 min after fatigue protocol (p < 0.001).

### Muscle elasticity

A significant main effects of *Intervention* (F_1,264_ = 613.0, *p* < 0.001, η^2^ = 0.90) and *Time* (F_1,68_ = 19.9, *p* < 0.001, η^2^ = 0.45), and a significant Interaction between *Intervention* × *Time* (F_3,69_ = 47.0, *p* = 0.001, η^2^ = 0.82) was showed. Post-hoc analysis revealed increased muscle elasticity from baseline to immediately and 30 min after fatigue protocol in all recovery procedures (*p* < 0.001 for all). Additionally, PICT at 100 mmHg showed greater elasticity compared to CCT at 30 min and 48 h after fatigue protocol (*p* < 0.001 for both). Further, CCT exhibited greater elasticity compared to the control group immediately after fatigue protocol (*p* < 0.001), and PICT at 100 mmHg showed greater elasticity compared to PICT at 25 mmHg 48 h after fatigue protocol (*p* < 0.001) (Fig. 5b).

### Lactate dehydrogenase activity (LDH)

A two-way mixed ANOVA revealed significant main effects of *Intervention* (F_3,44_ = 5.6, *p* = 0.002, η^2^ = 0.28) and *Time* (F_3,132_ = 1275.5, *p* < 0.001, η^2^ = 0.97), as well as a significant interaction between *Intervention* × *Time* (F_9,132_ = 3.7, *p* < 0.001, η^2^ = 0.20) on LDH levels. Post-hoc analysis showed that LDH increased from baseline to immediately and 30 min after fatigue protocol in all recovery strategies (*p* < 0.001 for all) (Fig. 5d).

### Reactive strength index (RSI)

RSI showed significant main effects of *Intervention* (F_3,44_ = 11.4, *p* < 0.001, η^2^ = 0.45) and *Time* (F_3,132_ = 284.3, *p* < 0.001, η^2^ = 0.85), as well as a significant interaction between *Intervention* × *Time* (F_9,132_ = 5.7, *p* < 0.001, η^2^ = 0.28). Post-hoc analysis revealed that RSI decreased from baseline to 30 min after fatigue protocol in CCT (*p* < 0.001) and 48 h after fatigue protocol in both PICT at 25 mmHg and 100 mmHg (*p* < 0.001 for all). Additionally, RSI was greater in CCT compared to PICT at 25 mmHg at 30 min after fatigue protocol. (*p* < 0.001), while RSI was lower in PICT at 25 mmHg compared to CCT and the control group 48 h after fatigue protocol (*p* < 0.001 for both) (Fig. 5e).

### Pressure pain threshold (PPT)

PPT showed a significant main effect of *Intervention* (F_3,264_ = 620, *p* < 0.001, η^2^ = 0.88), with no significant interaction of *Time* and *Intervention*. Overall, PPT decreased from baseline to 48 h after fatigue protocol in all recovery procedures (*p* < 0.001 for all) (Fig. 5c).

## Discussion

This study investigated the effects of different recovery strategies (PICT at 25 mmHg, PICT at 100 mmHg, and CCT) on post-exercise recovery. The findings of this study showed in line with the hypothesis, that both PICT at 100 mmHg and CCT increased tissue perfusion and muscle elasticity, while CCT showed greater perfusion at 30 min. Moreover, this study showed that all strategies reduced pain perception and muscle damage markers over time. Furthermore, PICT at 100 mmHg maintained the highest elasticity at 48 h. Muscle soreness decreased with CCT at 30 min and PICT (25 and 100 mmHg) at 48 h. In summary, the findings of the current study support the hypothesis that both pneumatic and cold-compression strategies may effectively increase post-exercise recovery by improving tissue and muscle regeneration.

To the best of our knowledge, this is the first RCT study focused on both pneumatic and cryo-compression in athletes’ recovery. Previously, studies evaluated separately these two therapy procedures, including intermittent compression and cryo-compression therapy after eccentric exercise^1,7,10,28^. In our study, the tissue perfusion increased immediately after fatigue protocol in all groups, except the control group. CCT and PICT at 100 mmHg showed the greatest increase in tissue perfusion at 30 min after fatigue protocol, compared to the control group and PICT at 25 mmHg. Recently published studies have demonstrated an increase in tissue perfusion after fatigue exercise and CCT recovery^1,9,29^. Our results confirm previous findings and also present interesting new insights into PICT. A greater increase in tissue perfusion at 100 mmHg may reflect greater blood circulation because of increased tissue fluid exchange^30^. Moreover, the greatest increase in tissue perfusion at 30 min after fatigue in CCT and PICT at 100 mmHg compared to PICT at 100 mmHg could be associated with increased microvascular dilation and improved blood flow dynamics^31^. It has been shown previously, that CCT may react on tissue perfusion in two phases, i.e., vasoconstriction and a decrease in tissue perfusion in the initial phase (approx. after 5 min) followed by hyperemic (approx. 24 h)^9^. Physiologically, CCT might be more beneficial than PICT. However, pneumatic compression, particularly PICT at 100 mmHg, can lead to a similar increase in tissue perfusion. One of the key physiological mechanisms of the regenerative processes after these recovery strategies is microcirculation^32^. It is crucial to maintain adequate blood flow to the injured tissue to facilitate the delivery of oxygen, nutrients, and growth factors, as well as the removal of metabolic waste products. After fatigue-induced muscle alterations it maintains adequate blood flow to the injured tissue to facilitate the delivery of oxygen, nutrients, and growth factors, as well as the removal of metabolic waste products^33^.

Muscle elasticity increased across all recovery methods immediately and 30 min post-fatigue. In particular, PICT at 100 mmHg demonstrated greater elasticity compared to CCT at 30 min and 48 h post-fatigue. These findings suggest that pneumatic compression, especially at higher pressures, may enhance muscle recovery. In other words, we observed a significant increase in muscle elasticity immediately following the recovery intervention, particularly with PICT at 100 mmHg. Our study indicates that compression contrast therapy is most effective in immediately restoring muscle elasticity. These changes in elasticity could be associated with a greater impact on muscle blood flow, which may lead to a reduction of muscle tension and simultaneously increase the elasticity^9,29^. Alterations in muscle mechanical properties may improve the body’s ability to absorb external forces and minimize energy consumption during movement. Optimal energy dissipation necessitates muscle bundles with suitable elastic properties, facilitating efficient strain elongation and recoil^34^. Research suggests that a complex mechanism depends on precise neural regulation and adequate blood supply to microcirculation^35,36^.

This study found that different recovery strategies significantly impacted muscle damage, as measured by LDH levels. Regardless of the recovery method, muscle damage increased immediately after exercise and remained elevated 30 min later. Milovančev et al. evaluated alterations in cardiac biomarkers following rapid weight loss and high-intensity training in combat sports signify physiological stress and muscle damage that need to be carefully monitored to optimize athlete health and recovery. However, the specific recovery strategy influenced the severity and duration of this damage. Previously, Trybulski, et al.^1^ found that combined contrast (heat-cold) pressure therapy may lead to faster decreases in LDH, as a result of an increase in lactate clearance, reducing post-exercise swelling, and improving blood flow to fatigued muscles^37^. This mechanism is thought to be related to the alternating vasoconstriction and vasodilation caused by temperature fluctuations, leading to increased blood flow and accelerated clearance of muscle damage markers^38^.

Finally, the RSI data revealed that different recovery strategies significantly influenced muscle soreness. While all strategies reduced soreness over time, CCT proved most effective immediately after, whereas PICT at 25 mmHg was more effective in reducing DOMS 48 h after fatigue-induced exercise. Trybulski, et al.^1^ reported that contrast therapy significantly improved muscle strength recovery, as measured by the RSI, at specific time points post-exercise. These observations are in line with our study, and observed findings may suggest beneficial effects for accelerating recovery and enhancing performance. Specifically, PICT may improve recovery, particularly when consumed at a higher pressure (100 mmHg). However, a CCT may be more effective for immediate post-exercise recovery^29^.

PPT significantly decreased from baseline to 48 h after the fatigue protocol in all recovery procedures. A decrease in PPT values indicates increased pain sensitivity. Thus, reductions observed across all groups suggest that full recovery of pressure pain threshold was not achieved within 48 h. While all recovery procedures were effective in reducing pain sensitivity, there was no significant difference in effectiveness between them. These results showed that all recovery strategies caused an increase in PPT and a simultaneous decrease in pain sensitivity. Moreover, none of the recovery strategies led to a return to baseline pain sensitivity. Yanaoka, et al.^39^ demonstrated an increase in PPT and a reduction of pain sensitivity after 20 min of intermittent pneumatic compression at 135 mmHg. Findings from this study suggest that PPT may be a mechanism associated with improved recovery^9,39^. On the other hand, CCT showed a similar effect to PICT. Ebell et al. (2017) found that intermittent cold stimuli may be more beneficial for muscle soreness than continuous CCT application. This suggestion is in line with Knobloch et al.^40^ and may suggest that a 10-min recovery using CCT results in decreased pain sensitivity.

### Physiological mechanisms

The use of both recovery strategies, i.e., PICT (especially at 100 mmHg) and CCT may provide a wide range of physiological mechanisms leading to regeneration of the musculoskeletal system. PICT may increase tissue perfusion as a response to greater blood flow to the altered muscle and soft tissue. Lukic-Sarkanovic et al. (2024) reported that rapid weight loss in wrestlers induces significant increases in markers of acute muscle damage, underscoring the metabolic stress imposed by weight-cutting practices and the necessity for targeted recovery interventions to mitigate muscle impairment. Furthermore, blood flow improves the removal of metabolic products and increases the delivery of oxygen and nutrients, accelerating tissue healing and simultaneously reducing inflammatory processes^31,41^. Furthermore, intermittent pneumatic compression reacts to muscle elasticity by reducing increased muscle tension and increasing blood flow^1^. Recent studies suggest that CCT induces initial vasoconstriction followed by vasodilation. This alternating cycle of blood flow restriction and release can improve blood flow to the affected area, reducing inflammation and accelerating tissue healing^42^.

### Limitations

This study has some limitations that should be pointed out. One limitation is the potential learning effect due to repeated measurements across time points. Additionally, the generalizability of findings may be limited to elite-level combat sport athletes and cannot be extended to amateur or recreational populations without caution. Although both male and female athletes were included, no subgroup analysis by sex was performed. Hormonal fluctuations related to the menstrual cycle may affect pain perception and vascular responses. This should be addressed in future studies. Additionally, studies should examine long-term effects of recovery strategies rather than only short-term outcomes. Lastly, using more advanced tools like shear-wave elastography or tensiomyography is recommended to better assess mechanical properties.

## Conclusions

Compression therapies (PICT and CCT) demonstrate effectiveness in improving tissue perfusion, muscle elasticity, pain perception, and muscle damage markers. PICT at 100 mmHg maintained the highest level of muscle elasticity at 48 h post-exercise, while CCT was most effective in reducing muscle soreness immediately after the fatigue-induced exercise protocol. These findings may suggest an optimal strategy for recovery until 48 h after exercise to maintain the return to sports activity. Future studies are needed to investigate a wide range of physiological and biomechanical outcomes for musculoskeletal system recovery. Among all evaluated outcomes, tissue perfusion and muscle elasticity emerged as the primary physiological indicators of recovery effectiveness, with RSI offering additional insight into functional readiness in athletes.

## Data Availability

Data used in this article can be obtained from the corresponding author at the reasonable request of a scientist.
